# The RNA-binding protein Pumilio limits polymodal nociception in *Drosophila* larvae

**DOI:** 10.7717/peerj.21481

**Published:** 2026-07-06

**Authors:** Sara Palega, Hannah Davenport, Rebeccah K. Stewart, Jessica Horton, Andrew Bellemer

**Affiliations:** 1 Biomedical Sciences Program, Eastern Virginia Medical School, Norfolk, Virginia, United States; 2 Department of Biology, Appalachian State University, Boone, North Carolina, United States; 3 Developmental Biology Department, Memorial Sloan Kettering Cancer Center, New York, New York, United States; 4 Department of Physics and Astronomy, Appalachian State University, Boone, North Carolina, United States

**Keywords:** *Drosophila*, Nociception, Gene regulation, Pumilio

## Abstract

Nociception is the process by which the nervous system detects and processes information about potentially harmful environmental stimuli to generate behavioral and physiological responses. The process of nociception undergoes plasticity in response to injury, inflammation, and infection to shape the sensitivity of nociceptive circuits and the behavioral responses they control. We are increasingly aware that post-transcriptional regulation of gene expression shapes the sensitivity and plasticity of nociceptive sensory neurons and nociceptive behavior. In *Drosophila*, the Pumilio RNA-binding protein interacts with diverse target mRNAs to regulate their abundance and translation in a wide range of contexts including the establishment of embryonic polarity, maintenance of the germline, regulation of neural morphology, and homeostatic control of neural excitability. In this study, we find that Pumilio acts to limit nociceptive sensitivity through its activity in nociceptive sensory neurons. Larvae with nociceptor-specific *pumilio* knockdown show enhanced nocifensive responses to noxious thermal, mechanical, and chemical stimuli, while larvae with nociceptor-specific overexpression of *pumilio* show blunted responses to noxious thermal and mechanical sensitivity. Furthermore, we find that Pumilio is required in the nociceptors for larvae to expression nociceptive sensitization following injury, suggesting a role for Pumilio in nociceptor plasticity. These observations may be explained by regulation of neuronal excitability, dendrite morphology, and cell signaling by Pumilio, and future studies will elucidate these mechanisms.

## Introduction

Nociception is the process by which the nervous systems of vertebrate and invertebrate animals detect potentially harmful environmental stimuli to generate appropriate behavior responses, such as escape behavior. In diverse animal taxa, tissue damage causes sensitization of primary nociceptor neurons and nociceptive neural circuits, leading to heightened nociceptive behavior (Babcock, Landry & Galko, 2009; Crook, Hanlon & Walters, 2013; Illich & Walters, 1997; Yang et al., 2014). Recent studies in cephalopods and rodents suggest that sensitization of nociceptive responses is an adaptive response that allows injured animals to avoid predation (Crook et al., 2014, 2011; Lister et al., 2020). However, it is also well understood that plasticity in nociceptors and nociceptive circuits underlie persistent pain states that often require clinical intervention (Reichling & Levine, 2009).

Injury-induced sensitization of nociceptors is associated with changes in gene expression regulated both transcriptionally and post-transcriptionally (Hayward et al., 2024; Khoutorsky & Price, 2018). For example, the translational regulator mammalian target of rapamycin (mTOR) is required for induction of persistent pain states and nociceptor sensitization, as inhibition of mTOR by administration of rapamycin prevents these phenomena (Asante, Wallace & Dickenson, 2009; Geranton et al., 2009). Likewise, phosphorylation of the mRNA cap-binding protein, eIF4E, and the poly-A tail-binding protein, PAPB, are also required for induction of injury-induced sensitization and hyperalgesia, presumably due to their role in regulating translation (Barragán-Iglesias et al., 2018; Mihail et al., 2019; Moy et al., 2018). Collectively, these studies indicate a layer of post-transcriptional regulation on nociceptor sensitivity and plasticity, but the extent and diversity of mechanisms involved in this type of regulation is not known.

The *Drosophila* RNA-binding protein, Pumilio, is a member of the PUF (Pumilio and FBF) family of proteins. PUF proteins have widespread roles in post-transcriptional regulation of gene expression, generally acting to reduce mRNA and protein levels of their targets by destabilizing mRNA and inhibiting translation (Blewett & Goldstrohm, 2012; Chritton & Wickens, 2011; Goldstrohm et al., 2006, 2007; Van Etten et al., 2012; Weidmann et al., 2014). In *Drosophila*, Pumilio is most well-known for its role in embryogenesis. During this process, Pumilio binds along with the zinc-finger protein, Nanos, to short sequence elements in the 3′ untranslated region (3′ UTR) of *hunchback* mRNA to repress expression of the Hunchback morphogen in the posterior pole of the embryo (Barker et al., 1992; Murata & Wharton, 1995; Wreden et al., 1997). Pumilio and Nanos collaborate similarly in the development and maintenance of the *Drosophila* germline by repressing expression of proteins that would lead to mitosis and differentiation (Asaoka-Taguchi et al., 1999; Forbes & Lehmann, 1998; Lin & Spradling, 1997). Pumilio also has postmitotic roles in regulating the function of the nervous system. In both mice and flies, Pumilio proteins bind voltage-gated sodium channel mRNAs to limit sodium channel expression and to homeostatically regulate neural excitability (Driscoll et al., 2013; Mee et al., 2004; Muraro et al., 2008). Pumilio and Nanos are also required for normal development of the Class III and Class IV multidendritic sensory neurons of *Drosophila* larvae, as neurons lacking Pumilio function exhibit defects in the outgrowth and maintenance of dendrites during late larval stages (Olesnicky, Bhogal & Gavis, 2012; Ye et al., 2004).

*Drosophila* larvae respond to noxious thermal and mechanical stimuli using Class IV multidendritic neurons that are present in each larval body segment and which possess dendrites that tile the larval body wall (Grueber, Jan & Jan, 2002; Hwang et al., 2007; Tracey et al., 2003; Zhong, Hwang & Tracey, 2010). The behavioral response of fly larvae to noxious stimuli is termed nocifensive escape locomotion (NEL), which consists of a series of lateral rolls around the long body axis (Tracey et al., 2003). Analysis of NEL behavior has enabled the identification of the mechanisms that transduce nociceptive stimuli including thermosensory and mechanosensory ion channels (Kim et al., 2012; Neely et al., 2011; Tracey et al., 2003; Zhong et al., 2012; Zhong, Hwang & Tracey, 2010). Similar studies have also identified multiple signaling mechanisms that regulate sensitization of nociceptive responses following injury. These include signaling by cytokines, growth factors, morphogens, and neuropeptides using receptors expressed in the Class IV multidendritic neurons (Babcock, Landry & Galko, 2009; Babcock et al., 2011; Follansbee et al., 2017; Im et al., 2015; Lopez-Bellido et al., 2019b). Although the process of nociceptive sensitization in *Drosophila* larvae presumably requires changes in gene expression, as sensitized nociceptive responses do not emerge until hours after injury, both the mechanisms that accomplish this and the ultimate targets of such regulation are unknown.

In a previously published genetic screen, nine RNA-binding protein genes were identified as producing reduced thermal nociception sensitivity when knocked down in the larval nociceptors (Dyson et al., 2025). Although this screen was not designed to identify hypersensitive thermal nociception phenotypes, it was noted that nociceptor-specific knockdown of *pumilio* produced a potential hypersensitive thermal nociception.

In this study, we investigate the role of Pumilio as a potential regulator of nociceptor function. To accomplish this, we have knocked down and overexpressed Pumilio in the Class IV multidendritic neurons and measured the resulting changes in behavioral responses to thermal, mechanical, and chemical nociceptive stimuli. We also analyze the role of Pumilio in nociceptor plasticity using nociceptor-specific knockdown larvae in an injury-induced sensitization model.

## Methods

### *Drosophila* stocks and husbandry

The *ppk1.9-GAL4; UAS-dicer2* line used for nociceptor-specific expression was obtained from the laboratory of Dr. Dan Tracey and was identical to the line used in previous nociception studies (Herman, Willits & Bellemer, 2018; Zhong, Hwang & Tracey, 2010). *UAS-pum-RNAi* lines used in these studies were obtained from the Bloomington *Drosophila* Stock Center (BDSC) and Vienna *Drosophila* Resource Center (VDRC). *UAS-pum* overexpression lines were obtained from the laboratory of Dr. Josh Dubnau (Chen et al., 2008). The *UAS-para-RNAi* line originated from the VDRC.

All larvae used in this study were raised on cornmeal-molasses medium (Nutri-Fly M; Genesee Scientific, El Cajon, CA, USA) at 25 °C on a 12:12 h light:dark cycle.

### Thermal nociception assays

Thermal nociception assays were conducted as previously described (Dyson et al., 2025; Herman, Willits & Bellemer, 2018). Wandering third-instar larvae were washed from vials into a glass petri dish using distilled water. A pinch of dry baker’s yeast was added to the dish to disrupt the surface tension of the water and then water was removed to leave a thin film on the plate. Larvae were allowed to recover on the plate until they resumed normal locomotion (~3 min). Larvae were then stimulated with custom-built thermal probe consisting of a soldering iron with a ~6 mm chisel tip plugged into a Variac Variable Transformer (Part No. ST3PN1210B) (ISE, Inc., Cleveland, OH, USA) to control the temperature. Temperature was monitored during the experiment using an IT-1E thermistor attached to the probe tip using thermal epoxy and a BAT-12 digital thermometer (Physitemp, Clifton, NJ, USA). Experiments in which the experimental group was hyposensitive compared to controls were conducted at 46 °C, which induces a fast baseline response in control larvae, providing appropriate headroom to detect a range of slower response latencies. Experiments in which the experimental group was hypersensitive were conducted at 42 °C, which induces a relatively slow baseline response in control larvae, providing us with the ability to detect a range of faster response latencies. Trials in which the probe temperature deviated more than 0.5 °C from the desired temperature were discarded from analysis. Larval genotypes were obscured during both testing and analysis. Trials were captured using a digital video camera mounted on a dissecting microscope at 30 frames per second. Videos were analyzed using Adobe Premier Pro to identify the frame at which the probe contacted the larva and the completion of a 360° roll, and these time points were used to calculate the response latency. Trials in which the larva did not roll within 10 s were scored as 11 s for analysis. Trials in which the probe broke contact with the larvae prior to either NEL execution or 10 s were excluded from analysis. Experimenters were blind to genotype during testing and scoring.

### Mechanical nociception assays

Mechanical nociception assays were conducted as previously described (Herman, Willits & Bellemer, 2018). Larvae were obtained using methods identical to those previously described for the thermal nociception assay. Von Frey filaments were constructed by affixing monofilament nylon fishing line (Stren Original Monofilament 8 lb. line, Part #1304152; Pure Fishing, Inc., Columbia, SC, USA) to a glass Pasteur pipette. Filaments were trimmed to lengths that would provide either ~30 millinewtons or ~50 millinewtons of force when depressed. The ~30 millinewton probes were used in experiments where the experimental group was more sensitive than control groups, as this force produces a low baseline response rate in control larvae, allowing us to detect a range of greater response rates. The ~50 millinewton probes were used in experiments where the experimental groups were less sensitive than control groups, as this force produces a higher baseline response rate in control larvae, allowing us to detect a range of lower response rates. Larvae were stimulated along their midline equidistant between their anterior and posterior ends, and NEL responses were scored as a binary variable to calculate the proportion of larvae that responded to the stimulus. At least 90 larvae were tested per genotype. Experimenters were blind to genotype during testing and scoring.

### Chemical nociception assays

Larvae were obtained using methods identical to those previously described for the thermal nociception assay. One larva at a time was then transferred to a dry petri dish with a paintbrush and allowed to resume locomotion. At this time, 2 µl of 2M HCl was dispensed onto the larva with a micropipette and 1 min of behavioral responses were recorded using a digital video camera mounted on a dissecting scope. Larvae were discarded at the end of each trial, and the petri dish was rinsed with distilled water and dried between trials. Videos were analyzed using Adobe Premier Pro to identify trials in which NEL occurred (with NEL defined as a complete 360° rotation around the long body axis) and in which writhing occurred (with writhing defined as multiple, sequential bodies bends to the left and right side) (Robertson, Tsubouchi & Tracey, 2013). At least 40 larvae were tested for each genotype. Response rates were presented as the proportion of larvae exhibiting either NEL or writhing. Experimenters were blind to genotype during testing and scoring.

### Nociceptor sensitization assay

Early-third instar larvae were washed from vials using distilled water into a glass petri dish. The petri dish was then chilled on ice until the larvae became immobile. An insect pin (Austerlitz, 0.10 mm) was then used to pierce the epidermis along the dorsal midline between the A3 and A5 body segments. Injured larvae were then moved to apple juice agar plates seeded with yeast past and incubated at 25 °C for 8 h. Sham-injured larvae were subjected to this entire process except for the insect pin injury. Injured and sham-injured larvae were recovered from apple juice agar plates after 8 h for thermal nociception assays (as described above). During nociception analysis, experimenters were blinded to both genotype and injury status of each group.

### Statistical analysis

All data were analyzed using the Estimation Statistics website and DABEST python package (Ho et al., 2019). For thermal nociception data, a two-sided permutation t-test was used to determine *P* values for comparison between experimental and control groups. Effect sizes were calculated for each comparison using Cliff’s delta along with a bootstrap 95% confidence interval (CI). Cliff’s delta is a non-parametric measure of effect size that provides a method for estimating the magnitude of differences between genotypes without making assumptions about the distribution of the data (Cliff, 1993). For mechanical and chemical nociception data, a two-sided permutation t-test was used to determine *p* values for comparison between experimental and control groups. Effect sizes were calculated for each comparison using unpaired mean differences along with bootstrap 95% CIs.

## Results

### Pumilio regulates thermal nociception in *Drosophila* larvae

**Figure 1:**
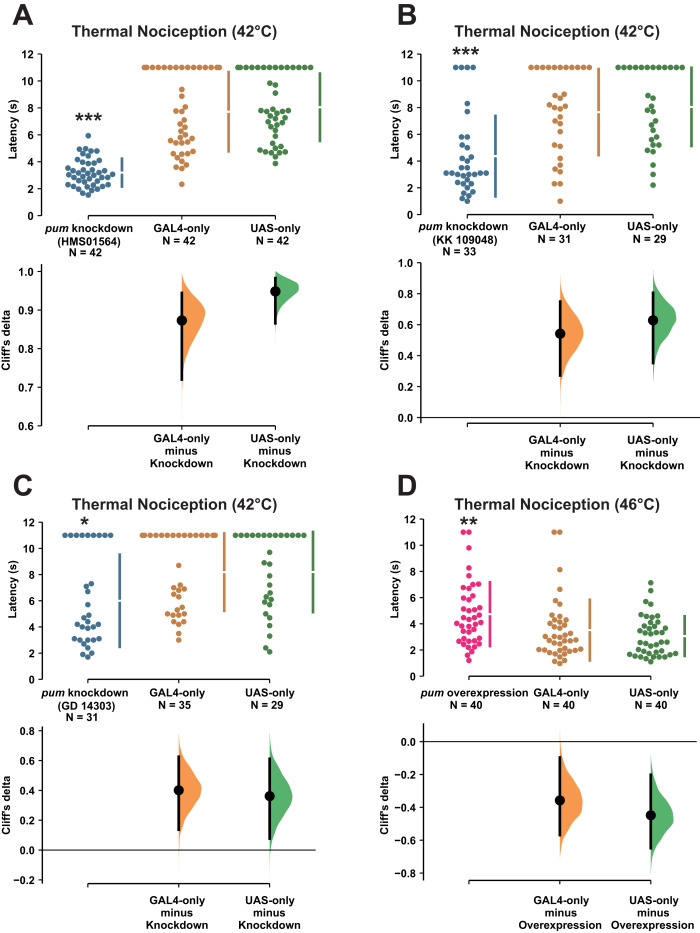
Pumilio regulates thermal nociception sensitivity in *Drosophila* larvae. (A–C) Nociceptor-specific knockdown of *pumilio* reduces the latency of NEL in response to a 42 °C thermal stimulus. In each panel, the top graph displays response latencies for *pum* knockdown and control larvae. The vertical bars to the right of each dot swarm display the mean latency value (indicated by the gap around the center of each bar) and standard deviation (indicated by the height of each bar on either side of the mean). In each panel, the bottom graph displays the Cliff’s delta effect-size measure for each comparison between the knockdown and control groups. These values are shown as a bootstrap sampling distribution with the mean difference represented by a dot and the 95% confidence interval represented by vertical bars. (D) Nociceptor-specific overexpression of *pumilio* increases the latency of NEL in response to a 46 °C thermal stimulus. This panel is organized in the same manner as the preceding panels. One asterisk (*) indicates *p* ≤ 0.01 compared to the GAL4-only control group and *p* ≤ 0.05 compared to UAS-only control group by permutation t-test. Two asterisks (**) indicate *p* ≤ 0.01 compared to the GAL4-only control group and *p* ≤ 0.005 compared to UAS-only control group by permutation t-test. Three asterisks (***) indicate *p* ≤ 0.0005 compared to the GAL4-only control group and UAS-only control group by permutation t-test.

To identify the role of Pumilio in regulating thermal nociception, we used UAS-RNAi transgenes in combination with the *ppk-GAL4* driver to knock down *pumilio* specifically in the Class IV multidendritic neurons. Knockdown larvae and GAL4-only and UAS-only controls were stimulated with a 42 °C thermal probe to produce NEL, allowing response latency to be compared between groups. In our first experiment using the TRiP *HMS01564 pum* RNAi transgene (Fig. 1A), GAL4-only control larvae showed a mean response latency of 7.7 s, while UAS-only control larvae showed a mean response latency of 8.0 s. *pumilio* knockdown larvae, in comparison, showed a significantly different mean response latency of 3.2 s. The Cliff’s delta effect size for the comparison between the knockdown and GAL4-only group was 0.87 with a bootstrap 95% CI [0.72–0.94]. The Cliff’s delta effect size comparison between the knockdown and UAS-only group was 0.95 with a bootstrap CI of 0.87 to 0.98. In a second experiment using the VDRC *KK 109048 pum* RNAi line, *pum* knockdown larvae showed a significantly shorter response latency (4.4 s) than GAL4-only (7.7 s) and UAS-only (8.0 s) controls (Fig. 1B). In this experiment, the Cliff’s delta for the experimental to GAL4-only comparison was 0.54 (95% CI [0.27–0.75]), while the Cliff’s delta for the experimental to UAS-only comparison was 0.63 (95% CI [0.35–0.81]). In a third knockdown experiment using the VDRC *GD 14303 pum* RNAi line, *pum* knockdown larvae showed a significantly shorter response latency (6.0 s) than GAL4-only (8.2 s) and UAS-only (8.2 s) controls (Fig. 1C). In this experiment, the Cliff’s delta for the experimental to GAL4-only comparison was 0.40 (95% CI [0.14–0.63]), while the Cliff’s delta for the experimental to UAS-only comparison was 0.36 (95% CI [0.08–0.61]).

To determine whether the effect of Pumilio expression on thermal nociception was bidirectional, we also used a UAS-*pumilio* cDNA transgene with a *ppk-GAL4* driver to overexpress Pumilio in the nociceptors. When tested for thermal nociception responses at 46 °C, Pumilio overexpression larvae showed a response latency of 4.7 s, which was significantly longer than latencies observed in GAL4-only (3.5 s) or UAS-only (3.1 s) controls (Fig. 1D). In this experiment, the Cliff’s delta for the experimental to GAL4-only comparison was −0.36 (95% CI [−0.57 to −0.10]), while the Cliff’s delta for the experimental to UAS-only comparison was −0.45 (95% CI [−0.65 to −0.20]).

These observations were somewhat unexpected, as previous studies have indicated a loss of nociceptor sensitivity in response to *pumilio* knockdown (Xu, Brechbiel & Gavis, 2013). To confirm our findings, which are consistent with heightened nociceptive sensitivity in *pum* knockdown larvae, we report confirmatory thermal nociception experiments that were conducted by an independent experimenter. The results of these experiments are contained in Table S1 and show similarly shortened NEL latency in *pum* knockdown conditions.

### Pumilio regulates mechanical nociception in *Drosophila* larvae

**Figure 2:**
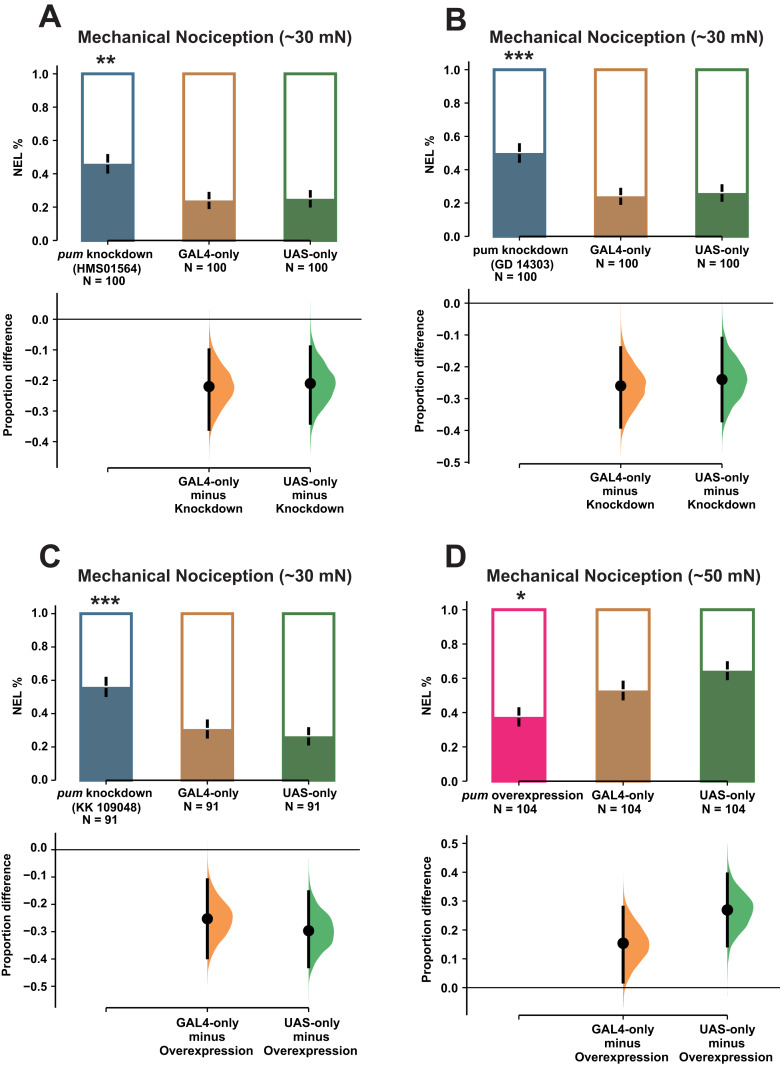
Pumilio regulates mechanical nociception sensitivity in *Drosophila* larvae. (A–C) Nociceptor-specific knockdown of *pumilio* increases the proportion of larvae that respond to a ~30 millinewton mechanical stimulus. In each panel, the top graph displays the proportion response data for each genotype, with the white part of the graph indicating non-responders and the filled part of the graph representing larvae that responded with NEL. The error bars indicate standard deviation. The bottom graph in each panel displays the proportion differences between the experimental and control groups. These values are shown as a bootstrap sampling distribution with the proportion difference represented by a dot and the 95% confidence interval represented by vertical bars. (D) Nociceptor-specific overexpression of *pumilio* decreases the proportion of larvae that respond to a ~50 millinewton mechanical stimulus. The graphs in this panel are organized the same as preceding panels. One asterisk (*) indicates *p* ≤ 0.05 compared to the GAL4-only control and *p* ≤ 0.0005 compared to the UAS-only control group by permutation t-test. Two asterisks (**) indicate *p* ≤ 0.005 compared to both the GAL4-only and UAS-only control groups. Three asterisks (***) indicate *p* ≤ 0.0005 compared to both the GAL4-only and UAS-only control groups.

To assay the role of Pumilio in mechanical nociception, we used the same nociceptor-specific knockdown approach as described above for thermal nociception. In these experiments, knockdown and transgenic control larvae were probed with a Von Frey filament and the proportions of animals that displayed an NEL response were used to compare the mechanical nociceptive sensitivity of different genotypes. In our first experiment using the TRiP *HMS01564 pum* RNAi transgene, we found that 46% of *pum* knockdown larvae showed NEL responses to the mechanical stimulus, while significantly smaller proportions of GAL4-only (24%) and UAS-only (25%) controls showed NEL responses (Fig. 2A). In this experiment, the mean difference between the knockdown and GAL4-only groups was −0.22 (95% CI [−0.36 to −0.1]), while the mean difference between the knockdown and UAS-only groups was −0.21 (95% CI [−0.34 to −0.09]). In a second knockdown experiment using the VDRC *GD 14303 pum* RNAi transgene, we found that 50% of *pum* knockdown larvae exhibited an NEL response to Von Frey stimulation, while significantly smaller proportions of GAL4-only (24%) and UAS-only (26%) controls exhibited NEL responses (Fig. 2B). In this experiment, the mean difference between the knockdown and GAL4-only groups was −0.26 (95% CI [−0.39 to −0.14]), while the mean difference between the knockdown and UAS-only groups was −0.24 (95% CI [−0.37 to −0.11]). In a third knockdown experiment using the VDRC *KK 109048 pum* RNAi line, we found that 56% of *pum* knockdown larvae exhibited NEL responses, while significantly smaller proportions of GAL4-only (31%) and UAS-only (26%) controls did the same (Fig. 2C). In this experiment, the mean difference between knockdown and GAL4-only group was −0.25 (95% CI [−0.40 to −0.11]), while the mean difference between the knockdown and UAS-only group was −0.30 (95% CI [−0.43 to −0.15]).

To test whether overexpression of Pumilio would have an opposite effect to that of knockdown, we also performed mechanical nociception assays on larvae with nociceptor-specific overexpression of Pumilio along with GAL4-only and UAS-only controls. In this experiment, 37.5% of Pumilio overexpression larvae exhibited NEL responses to Von Frey stimulation, while a significantly larger proportions of GAL4-only (52.9%) and UAS-only (64.4%) larvae responded (Fig. 2D). In this experiment, the mean difference between the knockdown and GAL4-only groups was 0.15 (95% CI [0.02–0.28]), while the mean difference between the knockdown and UAS-only groups was 0.27 (95% CI [0.14–0.39]).

### Pumilio regulates chemical nociception in *Drosophila* larvae

**Figure 3:**
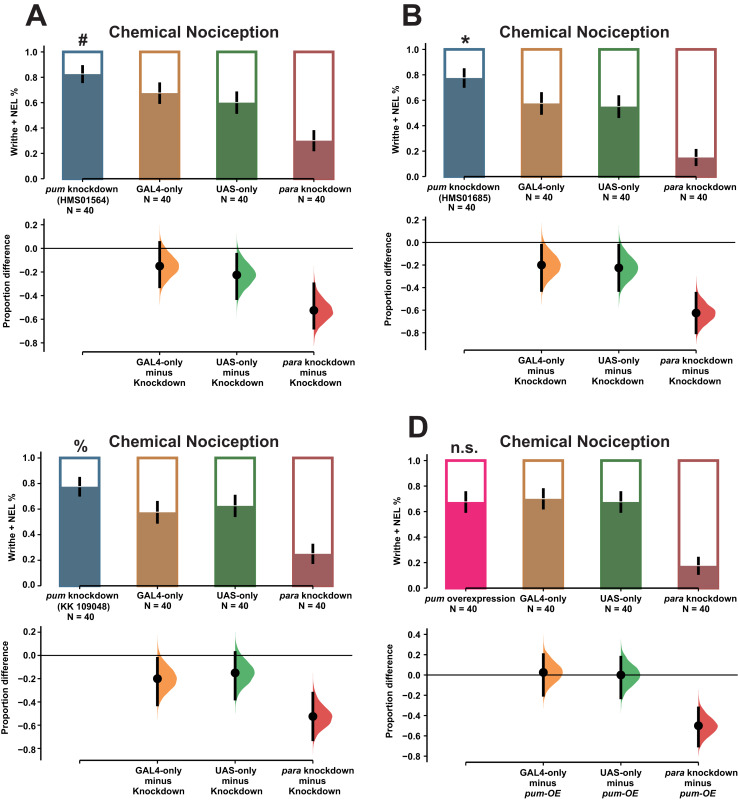
Pumilio regulates chemical nociception sensitivity in *Drosophila* larvae. (A–C) Nociceptor-specific knockdown of *pumilio* increases the proportion of larvae that respond to 2M HCl with nociceptive writhing or NEL responses. In each panel, the top graph displays the proportion response data for each genotype, with the white part of the graph indicating non-responders and the filled part of the graph representing larvae that responded with writhing or NEL. The error bars indicate standard deviation. The bottom graph in each panel displays the proportion differences between the experimental and control groups. These values are shown as a bootstrap sampling distribution with the proportion difference represented by a dot and the 95% confidence interval represented by vertical bars. (D) Nociceptor-specific overexpression of *pumilio* does not have a significant effect on writhing or NEL responses. The graphs in this panel are organized the same as preceding panels. # indicates *p* ≤ 0.05 in comparison to the UAS-only control group and *p* > 0.05 in comparison to the GAL4-only control group by permutation t-test. One asterisk (*) indicates that *p* ≤ 0.05 in comparison to GAL4-only and UAS-only control groups. % indicates *p* ≤ 0.05 in comparison to the GAL4-only control group and *p* > 0.05 in comparison to the UAS-only control group by permutation t-test.

We also tested how *pumilio* expression controls responses to noxious chemical stimuli. In these experiments, nociceptor-specific *pum* knockdown larvae were exposed to 2 µl of 2M HCl in a plastic petri dish, while nociceptive behaviors including nociceptive writhing and NEL were scored. In a knockdown experiment using the TRiP *HMS01564 pum* RNAi transgene, 82.5% of *pumilio* knockdown larvae displayed either nociceptive writhing or NEL following HCl exposure, which was significantly greater than the proportion UAS-only (60%) larvae that exhibited similar responses, but not significantly greater than the proportion of responses in GAL4-only larvae (67.5%) (Fig. 3A). An additional control group with nociceptor-specific knockdown of the *para* voltage-gated sodium channel mRNA, a manipulation which should largely silence action potentials in the nociceptors, showed a 30% response rate. In this experiment, the mean difference between the knockdown and GAL4-only control group was −0.15 (95% CI [−0.325 to 0.05]) and the mean difference between the knockdown and UAS-only group was −0.23 (95% CI [−0.43 to −0.05]). In a second experiment using the TRiP *HMS01685 pum* RNAi transgene, 77.5% of *pumilio* knockdown larvae showed nociceptive writhing or NEL responses to HCl, while significantly smaller proportions of GAL4-only (57.5%) and UAS-only (55%) larvae showed similar responses (Fig. 3B). An additional control group with nociceptor-specific knockdown of the *para* voltage-gated sodium channel mRNA showed a 15% response rate. In this experiment, the mean difference between the knockdown and GAL4-only control group was −0.2 (95% CI [−0.43 to −0.03]) and the mean difference between the knockdown and UAS-only group was −0.23 (95% CI [−0.43 to −0.03]). In a third knockdown experiment using the VDRC *KK 109048 pum* RNAi line, 78% of *pum* knockdown larvae showed nociceptive responses to HCl (Fig. 3C). This was significantly greater than the proportion of responding GAL4-only larvae (55%), but not the proportion of responding UAS-only control larvae (62.5%). In this experiment, the mean difference between knockdown and GAL4-only larvae was −0.23 (95% CI [−0.43 to −0.03]), while the mean difference between knockdown and UAS-only control larvae was −0.15 (95% CI [−0.38 to 0.03]). When Pumilio overexpression larvae were tested in this paradigm, no significant differences were observed between the knockdown group, GAL4-only group, and UAS-only group (Fig. 3D).

### Wild-type levels of Pumilio expression are required for injury-induced nociceptor-plasticity in *Drosophila* larvae

**Figure 4:**
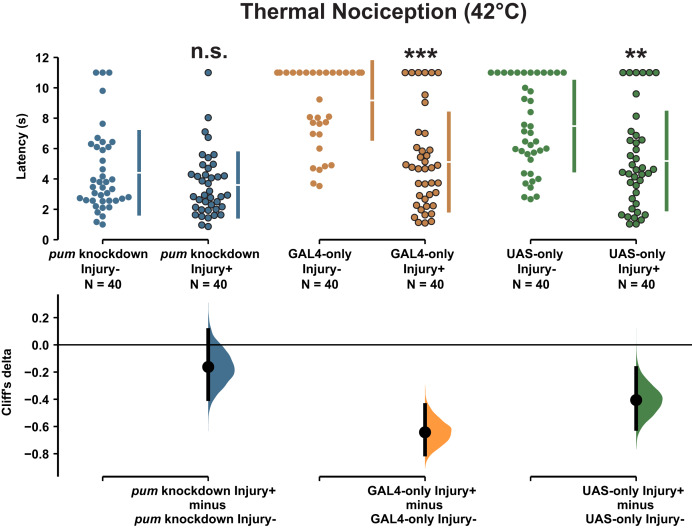
Wild-type levels of Pumilio expression are required for injury-induced hyperalgesia in *Drosophila* larvae. Injured GAL4-only and UAS-only control larvae show reduced thermal nociception response latency compared to sham-injured controls, while injured *pumilio* knockdown larvae (*HMS01564*) do not show sensitized responses. The top portion of the graph displays NEL latency data for knockdown and control larvae in injured and sham-injured conditions. The vertical bars to the right of each dot swarm display the mean latency value (indicated by the gap around the center of each bar) and standard deviation (indicated by the height of each bar on either side of the mean). The bottom graph shows the Cliff’s delta effect size measure for comparisons between injured and sham-injured groups. Two asterisks (**) indicate *p* ≤ 0.005 by permutation t-test comparison of injured and sham-injured groups. Three asterisks (***) indicate *p* ≤ 0.0005 by permutation t-test comparison of injured and sham-injured groups.

*Drosophila* larvae exhibit hyperalgesia responses following tissue damage, and this effect arises in part from signaling in the Class IV multidendritic neurons (Babcock, Landry & Galko, 2009; Im et al., 2015). To test whether Pumilio is required for normal nociceptive sensitization, we conducted an experiment to measure injury-induced hyperalgesia in nociceptor-specific *pumilio* knockdown and control larvae. Thermal nociception latencies to a 42 °C stimulus were measured for GAL4-only and UAS-only control larvae that were either injured with an insect pin or received a sham-injury protocol. In both genotypes, the injured larvae exhibited significantly shorter thermal nociception response latencies than their sham-injured controls (5.1 s *vs*. 9.2 s for GAL4-only larvae; 5.2 s *vs*. 7.5 s for UAS-only larvae) (Fig. 4). The Cliff’s delta effect size for uninjured *vs*. injured GAL4-only controls was −0.64 (95% CI [−0.81 to −0.44]), while the Cliff’s delta for uninjured *vs*. injured UAS-only controls was −0.41 (95% CI [−0.62 to −0.17]). When nociceptor-specific *pumilio* knockdown larvae (*HMS01564*) were tested under the same conditions, there was not a statistically significant latency difference observed between injured larvae and sham-injured controls (3.6 s *vs*. 4.4 s). The Cliff’s delta effect size for sham-injured knockdown larvae *vs*. injured knockdown larvae was −0.16 (95% CI [−0.40 to 0.11]). This indicates that larvae with nociceptor-specific *pum* knockdown fail to undergo nociceptive sensitization following injury.

## Discussion

In these experiments, nociceptor-specific knockdown of *pumilio* led to heightened nociceptive responses to noxious thermal, mechanical, and chemical stimuli. Conversely, nociceptor-specific overexpression of *pumilio* led to reduced sensitivity to noxious thermal and mechanical stimuli. These results suggest that Pumilio generally acts to limit nociceptor sensitivity under baseline conditions and that nociceptor sensitivity is inversely correlated with Pumilio expression.

The mechanism by which Pumilio might regulate nociceptor sensitivity has not been identified. One possibility is that Pumilio regulates nociceptor function at the level of electrical excitability and neurotransmission. Pumilio has been well-established as a homeostatic regulator of neural excitability *via* its regulation of sodium channel mRNA in both flies and vertebrates (Driscoll et al., 2013; Mee et al., 2004; Muraro et al., 2008). The Para voltage-gated sodium channel is the primary voltage-gated sodium channel encoded in the *Drosophila* genome, and its function is required in the nociceptors to establish baseline sensitivity to noxious thermal, mechanical, and chemical stimuli (Zhong, Hwang & Tracey, 2010). The *para* mRNA is also a target of negative regulation by Pumilio *via* direct binding within the para open-reading frame (Muraro et al., 2008). This suggests a hypothesis that Pumilio binds *para* mRNA in the nociceptors to negatively regulate channel expression and nociceptor excitability. This study does not provide measure nociceptor gene expression, but future studies could test this hypothesis by analyzing Para channel expression in the Class IV multidendritic neurons using fluorescent reporters or electrophysiological methods. It should be noted that Pumilio directly or indirectly regulates additional genes related to neurotransmission, including ionotropic glutamate receptor genes in flies and vertebrates and the *Drosophila* PSD95 homolog gene, *discs large* (Chen et al., 2008; Fiore et al., 2014; Menon et al., 2009, 2004). These may also contribute to neurotransmission changes in the nociceptors in response to Pumilio manipulation.

Pumilio may also regulate nociception through its function in nociceptor dendrite morphogenesis. Along with the RNA-binding protein Nanos (Nos), Pumilio regulates the dendritic arborization of the nociceptors in part through negative regulation of the proapoptotic factor, Hid, and positive regulation of the transcription factor, Cut (Bhogal, Plaza-Jennings & Gavis, 2016; Olesnicky, Bhogal & Gavis, 2012; Ye et al., 2004). In third-instar nociceptors lacking either Pumilio or Nanos, dendrite stabilization is defective, leading to a loss of terminal branches (Olesnicky, Bhogal & Gavis, 2012). More severe loss of dendrite branches and length is observed in nociceptors overexpressing either Pumilio or Nanos (Ye et al., 2004). It is possible that these morphological phenotypes underly the behavioral consequences of manipulating Pumilio expression. However, there does not appear to be a direct relationship between the number of terminal branches in the nociceptors and their sensitivity to noxious stimuli, as *pumilio* knockdown results in fewer dendritic branches, but increased nociceptor sensitivity. Thus, there is a clear need for additional studies that investigate the relationship between the morphological dynamics of the Class IV multidendritic neurons and their nociceptive function.

In addition to controlling baseline nociceptor sensitivity, Pumilio also appears to play a role in regulating nociceptor plasticity. Larvae with nociceptor-specific *pumilio* knockdown do not exhibit injury-induced hyperalgesia under conditions where hyperalgesia is observed in control larvae, suggesting that further sensitization is not possible when Pumilio expression is clamped at a low level. One possible interpretation of this observation is that Pumilio is upstream or downstream of one or more of the signaling pathways that regulate injury-induced nociceptor sensitization in *Drosophila* and that baseline negative regulation of nociceptor sensitivity by Pumilio is relieved as part of the normal mechanism of nociceptor sensitization. Thus, larvae with *pumilio* knockdown are essentially sensitized in the absence of injury, and injury is not able to further enhance sensitization. However, it is also possible that the lack of sensitization in *pumilio* knockdown larvae represents a ceiling effect, as *pumilio* knockdown larvae already display response latencies considerably faster than control larvae. Thus, further sensitization by injury is not possible, even if normal sensitization mechanisms still function in the absence of Pumilio. It is likely that future studies investigating the temporal dynamics of nociceptor sensitization under *pumilio* knockdown conditions as well as genetic interactions between *pumilio* and other regulators of nociceptor sensitization would be fruitful. For example, the BMP signaling pathway regulates injury-induced nociceptor sensitization (Follansbee et al., 2017; Gjelsvik, Follansbee & Ganter, 2018; Honjo & Tracey, 2018). In *Drosophila* germline contexts, BMP signaling is attenuated by the function of Pumilio (Harris et al., 2011; Newton et al., 2015). This suggests a test case for how Pumilio might interact with existing nociception regulatory mechanisms.

Within the broader goal of understanding mechanisms of nociception regulation, this study identifies a previously unknown role for a post-transcriptional regulator in controlling nociceptive sensitivity. Previous studies have identified roles for the mRNA cap-binding protein, eIF4E, in nociceptor plasticity in mice and *Aplysia* (Mihail et al., 2019; Moy et al., 2017, 2018). The RBP CUG triplet repeat binding protein (CUGBP) embryonic lethal abnormal vision (Elav)-like family member 4 (CELF4) has also recently been identified as a negative regulator of nociception in a mouse model (Mueth et al., 2025). Combined with previous studies, our results suggest that post-transcriptional regulation is a general mechanism for regulating nociceptor function across taxa. Future studies may address possible combinatorial regulation by these individual mechanisms.

Two of our findings may warrant additional investigation. First, we find that reduced Pumilio expression enhanced nociceptor sensitivity in all knockdown genotypes and sensory modalities tested. This is not consistent with a previous finding that a *pumilio* mutant and *pumilio* knockdown cause reduced sensitivity to noxious mechanical stimuli (Xu, Brechbiel & Gavis, 2013). We were unable to identify the source of this discrepancy, but one possibility is that previous studies have used a relatively strong mechanical stimulus that produces a >70% response in control genotypes. It is possible that the effect of *pumilio* knockdown is not uniform across the entire range of stimulus intensities that the nociceptors detect (although we did not find evidence of this type of effect). We also note that the effect of *pumilio* knockdown and overexpression on chemical nociception was smaller than effects on mechanical and thermal nociception. A possible explanation for this is that thermal and mechanical nociception responses can be attributed almost entirely to the Class IV multidendritic neurons (Hwang et al., 2007; Zhong, Hwang & Tracey, 2010), whereas responses to HCl may require additional classes of multidendritic dendritic arborization neurons (Lopez-Bellido et al., 2019a). Our studies use a Class IV-specific GAL4 transgene. So, it is possible that we would observe stronger effects on chemical nociception if we were to knock down *pumilio* in all classes of multidendritic neurons. This requirement for additional classes of multidendritic neurons in HCl sensing may also explain why knockdown of the voltage-gated sodium channel mRNA, *para*, was insufficient to completely eliminate nociceptive responses to HCl, given that this manipulation almost completely eliminates thermal and mechanical nociception responses (Herman, Willits & Bellemer, 2018).

## Conclusions

This study demonstrates an important role for the RNA-binding protein, Pumilio, in the regulation of polymodal nociception and nociceptor sensitization. Nociceptor-specific knockdown of Pumilio produced heightened thermal, mechanical, and chemical nociceptive behavior, while Pumilio overexpression led to blunted thermal and mechanical nociception responses. Pumilio was also required for nociceptor plasticity, as nociceptor-specific knockdown of Pumilio prevented injury-induced hyperalgesia, although it remains possible that this is a ceiling effect. The mRNA target(s) of Pumilio in its regulation of nociception remain unknown, but previous studies have strongly indicated that Pumilio negatively regulates the voltage-gated sodium channel, Para, in motor neurons. Although we do not measure gene expression in these studies, our data are consistent with an inverse relationship between Pumilio and Para expression, suggesting that similar regulation of voltage-gated sodium channels by Pumilio may occur in nociceptors.

## Supplemental Information

10.7717/peerj.21481/supp-1 Supplemental Information 1 Raw data. Includes nociceptive response latencies for thermal nociception experiments and nociception response frequencies for mechanical and chemical nociception experiments.

10.7717/peerj.21481/supp-2 Supplemental Information 2 Confirmatory experiments indicate a role for Pumilio in thermal nociception.

## Additional Information and Declarations

### Data Availability

The following information was supplied regarding data availability:

The data is available in the Supplemental Files.
